# Progress and Recent Developments in HIV Vaccine Research

**DOI:** 10.3390/vaccines13070690

**Published:** 2025-06-26

**Authors:** Iris Shim, Lily Rogowski, Vishwanath Venketaraman

**Affiliations:** College of Osteopathic Medicine of the Pacific, Western University of Health Sciences, Pomona, CA 91766, USA; iris.shim@westernu.edu (I.S.); lily.rogowski@westernu.edu (L.R.)

**Keywords:** HIV, vaccines, vaccine efficacy

## Abstract

Background: Human immunodeficiency virus (HIV) remains a global health challenge despite significant advancements in antiretroviral therapy and prevention strategies. Developing a safe and effective vaccine that protects people worldwide has been a major goal, yet the genetic variability and rapid mutation rate of the virus continue to pose substantial challenges. Methods: In this review paper, we aim to provide a comprehensive review of previous vaccine candidates and the progress made in HIV vaccine clinical trials, spanning from the late 1990s to 2025. PubMed and ClinicalTrials.gov were searched for English-language Phase 1–3 HIV vaccine trials published from 1990 to March 2025. After de-duplication, titles/abstracts and then full texts were screened; trial phase, regimen, immunogenicity, efficacy, and correlates were extracted into a structured spreadsheet. Owing to platform heterogeneity, findings were synthesized narratively and arranged chronologically to trace the evolution of vaccine strategies. Results: Early vaccine trials demonstrated that a protein subunit vaccine failed to protect against infection, revealing the complexity of HIV evasion strategies and shifting the focus to a comprehensive immune response, including both antibody and T-cell responses. Trials evaluating the role of viral vectors in generating cell-mediated immunity were also insufficient, and suggested that targeting T cell response alone was not enough. In 2009, the RV144 trial made a breakthrough by showing partial protection against HIV infection and providing the first indication of efficacy. This partial success influenced subsequent trials, prompting researchers to further explore the complex immune response required for protection and consider combinations of vaccine technologies to achieve robust, long-lasting immunity. Conclusion: Despite setbacks, decades of rigorous efforts have provided significant contributions to HIV vaccine discovery and development, offering hope for preventing and protecting against HIV infection. The field remains active by continuing to advance our understanding of the virus, refining vaccine strategies, and employing novel technologies.

## 1. Introduction

Human immunodeficiency virus (HIV) remains one of the world’s most challenging infectious diseases, with 1.5 million new infections and 680,000 deaths in 2020 alone [1]. While antiretroviral therapy and preventive measures like pre-exposure prophylaxis (PrEP) have significantly reduced HIV mortality and transmission, they have not halted the pandemic [1]. A safe and effective HIV vaccine is urgently needed to achieve durable control of the epidemic [2]. Developing such a vaccine has proven extraordinarily difficult due to HIV’s tremendous genetic diversity, its ability to evade immune detection, and the lack of any cases of natural sterilizing immunity to use as a guide [2,3].

Research into HIV vaccines began in the late 1980s, and over the past three decades, numerous vaccine strategies have been tested [4]. Early approaches in the 1990s focused on inducing antibody responses with recombinant HIV envelope glycoprotein subunit vaccines (e.g., the AIDSVAX gp120 vaccines), but the first Phase III trials (VAX004 in North America/Europe and VAX003 in Thailand) showed no protective efficacy [3]. Subsequent T-cell vaccine strategies in the 2000s used viral vectors, such as adenovirus type 5, to induce robust CD8^+^ T-cell immunity; however, the STEP trial from 2004 to 2007 failed to prevent infection or reduce viral loads and even showed a higher infection rate in certain subgroups of vaccine recipients [3]. A turning point came in 2009 with the RV144 trial in Thailand—a heterologous prime-boost regimen using a canarypox vector (ALVAC) plus gp120 protein, which demonstrated a modest 31.2% reduction in HIV acquisition risk [4]. RV144 provided the first proof-of-concept that an HIV vaccine could protect humans, spurring a new wave of trials to improve upon this result [4]. Since then, major efficacy trials and multiple Phase 1/2 studies have been conducted evaluating diverse platforms (DNA, viral vectors, protein subunits, mRNA vaccines) and novel immunogens (mosaic antigens, stabilized envelope trimers, etc.), alongside passive immunization approaches using broadly neutralizing antibodies (bnAbs) [5]. This review provides a comprehensive update (1990–2025) on these developments in HIV vaccine research, with a focus on the immunological mechanisms targeted, key clinical trials and their outcomes, recent insights into correlates of protection, and new vaccine design strategies.

## 2. Materials and Methods

We searched PubMed/MEDLINE and ClinicalTrials.gov for human HIV-1/2 vaccine studies published between 1 January 1990 and 31 March 2025. The final search was run on 15 May 2025 using the following text words (truncated as appropriate) and trial identifiers: Arduino “HIV vaccine” OR “HIV vaccine clinical trial” OR “HIV vaccine efficacy” OR “DNA HIV vaccine” OR “mRNA HIV vaccine” OR “broadly neutralizing antibodies HIV prevention” OR “HIV life cycle” OR “latent HIV” OR “HIV subtype*” OR RV144 OR HVTN 702 OR Imbokodo. Search strings were adapted to each database’s syntax. Reference lists of key reviews and trial reports were also scanned to capture any additional pivotal studies. Eligibility criteria included:

• Population: human participants, HIV-negative or living with HIV.
• Intervention: any prophylactic or therapeutic HIV vaccine (all platforms).
• Study design: Phase 1, 2, or 3 clinical trials (randomized or non-randomized).
• Language: English.

Preclinical studies were excluded unless they directly informed a linked clinical design.

Titles and abstracts were screened by the first author. Full texts were retrieved for potentially relevant records; duplicates and interim analyses of the same trial were removed. Reasons for excluding full-text articles (e.g., non-human, protocol only) were noted.

For each included study, we recorded the trial phase, location, population size, vaccine regimen (immunogen, platform, adjuvant, dosing), immunogenicity assays, efficacy outcomes (HIV incidence or viral-load change), and reported correlates of protection. Data were entered into a structured spreadsheet and cross-checked by a second author.

Because vaccine platforms and outcome measures varied widely, results were synthesized narratively and presented chronologically (1990 → 2025). Large Phase 2b/3 efficacy trials receive detailed discussion; smaller Phase 1/2 studies are highlighted when they introduced novel concepts or platforms. A summary table compares all included trials by regimen and key outcomes.

## 3. HIV Genome and Immunological Challenges

### 3.1. Genome

HIV is a member of the Retroviridae family and specifically targets CD4^+^ T cells, which are a critical component of the immune system and are responsible for coordinating an immune response [6]. The virus contains a positive-sense, single-stranded RNA genome that encodes various genes that facilitate the replication cycle. The Gag gene encodes structural proteins, the Pol gene encodes reverse transcriptase, integrase, and protease, which are critical enzymes for viral replication, and the Env gene encodes envelope glycoproteins [6]. Specifically, gp120 and gp41 are critical for facilitating the entry of the virus into the host cell. Gp120 binds to the CD4 receptor on the host cell while Gp41 mediates the fusion of the viral envelope with the host cell membrane [6,7]. The gp120 protein is complex, containing both conserved (C1–C5) and variable regions (V1–V5) [8]. The variable loops of the HIV envelope glycoprotein are more prone to mutation and differ in terms of loop length and amino acid composition [8]. This variability is critical to understanding the ability of HIV to avoid immune recognition and adapt. Of the five regions, the V1V2 region is the most diverse in terms of sequence length and composition and is hypothesized to modulate how the virus interacts with CD4 and the coreceptor [8,9]. Additionally, this region is able to shield certain epitopes, thus preventing the immune system from detecting the virus and facilitating infection [9]. The V3 loop, however, has been widely studied and is understood to be the main determinant of which coreceptor the virus attaches to [8]. The genome also encodes accessory proteins (vif, vpr, and vpu) and regulatory proteins (Tat and Rev) that are involved in modulating the host’s immune response and regulating gene expression [6,7].

Human immunodeficiency virus entry into the host cells involves viral glycoprotein Gp120 binding to a CD4 receptor on the host cell surface and is further facilitated through binding to a coreceptor on the host cell, either CCR5 or CXCR4 [6,7]. Coreceptor binding induces a conformational change that exposes Gp41 and allows the virus to fuse with the host cell membrane [6,7]. After entering the host, the viral RNA genome is transcribed into DNA by reverse transcriptase, allowing the viral genome to replicate and integrate itself into the host [7]. The integration of the virus eventually leads to the destruction of CD4^+^ T cells and, therefore, weakens the immune system overall.

There are two lineages of the virus, HIV-1 and HIV-2, with HIV-1 being more prevalent and accounting for 95% of infections globally [10]. HIV-1 is composed of four groups: main, outlier, non-M, and P viruses [11]. The main group is further divided into nine distinct subtypes: A, B, C, D, F, G, H, J, and K [11,12]. Subtype E is classified as a circulating recombinant form and results from the coinfection or superinfection of different subtypes [8]. In general, subtypes differ genetically as well as in their geographical distribution, with subtype C being the most prevalent and accounting for about 50% of infections worldwide [13].

### 3.2. Challenges

The development of an HIV vaccine has faced various challenges, with the most significant obstacles being genetic variability and high mutation rates. During its viral life cycle, HIV replicates using reverse transcriptase, which lacks proofreading capabilities, and as a result, errors made during viral replication are not detected or corrected [12]. Errors accumulate over time, significantly contributing to the high frequency of mutations and rapid evolution of HIV [14]. The emergence of viral variants also makes it difficult for the immune system to effectively recognize the virus, allowing it to evade the immune system and persist in the body [15]. The low level of helper T cells, which is characteristic of HIV infection, further impairs the body’s ability to produce an effective immune response [6]. HIV specifically targets CD4^+^ T lymphocytes, which secrete cytokines that activate other immune cells such as macrophages and dendritic cells [6,7].

Another major challenge to eradicating HIV is latent infection, which allows the virus to evade the host immune system [15]. Latent reservoirs are established when HIV integrates its genome into memory CD4^+^ T cells, which are not actively dividing but remain in the body [15]. The virus is dormant and does not actively replicate within the host; therefore, the host does not detect the virus in this state, yet it still has the potential to become reactivated and produce new viral particles [15]. Finally, the distinct subgroups of HIV demonstrate the extensive genetic variability that exists among different geographical regions and populations, which poses another obstacle to developing a universal vaccine [11]. Additionally, an individual who is already infected with HIV may be exposed to another variant, potentially resulting in coinfection with a new, distinct variant. The presence of multiple subtypes of HIV increases genetic diversity within an individual, further complicating treatment and vaccine development [11].

## 4. Clinical Trials Review

### 4.1. Early Preventive Vaccine Efficacy Trials (1990s–2003)

During early trials, vaccines used Gp120 as an antigen due to its crucial role in the viral entry. Two important Phase III clinical trials that highlighted the complexity of HIV vaccine development were VAX004 and VAX003 [3]. The first efficacy trials of HIV vaccines in humans tested simple monomeric gp120 protein subunit vaccines developed by VaxGen (AIDSVAX vaccines). VAX004 was a Phase 3 trial (1998–2003) in North America and Europe, enrolling ~5400 men who have sex with men (MSM) and high-risk women, testing a bivalent gp120 (subtype B/B) vaccine with alum adjuvant. VAX003 was a parallel Phase 3 in Thailand among injection drug users, using a subtype B/E gp120 with alum [3]. Both trials showed no efficacy in preventing HIV infection—the infection rates were essentially the same in vaccine and placebo groups [3]. No impact on viral load or disease progression was observed in those who did become infected [3,16]. These outcomes were a major setback, indicating that the gp120 antibodies induced (mostly non-neutralizing and strain-specific) were insufficient to protect against infection. However, a post hoc finding from VAX004 found that a subset of vaccine recipients with high antibody levels against V3 loop epitopes had slightly lower infection rates, hinting that antibody quality matters (though this was not conclusive) [3]. The failure of AIDSVAX alone shifted research focus towards prime-boost strategies and T-cell vaccines.

### 4.2. T-Cell–Focused Trials: STEP and Phambili (2004–2010)

In the early 2000s, the focus turned to using viral vectors to elicit strong cellular immunity, given that antibodies from prior vaccines were not broadly neutralizing enough. The STEP trial (HIV Vaccine Trial Network, HVTN 502, 2004–2007) was a Phase 2b clinical trial designed to test whether a viral vector-based recombinant adenovirus type 5 vector (rAd5) vaccine encoding internal HIV proteins (gag, pol, nef) could prevent HIV infection by eliciting a robust CD8^+^ T cell response [3]. STEP enrolled ~3000 participants (mostly MSM in the Americas and Australia). As expected, the Merck rAd5 vaccine induced potent T-cell responses, but the trial was stopped early when an interim analysis showed no efficacy: 24 infections occurred in the vaccine group vs. 21 in the placebo [3]. More troubling, subgroup analysis found higher HIV infection rates in vaccinated men who had pre-existing Ad5 immunity and were uncircumcised [3].

Follow up studies suggest that a potential mechanism for this is that pre-existing immunity to adenovirus serotype 5 (Ad5) from natural infections caused the Ad5-based HIV vaccine to stimulate memory responses to Ad5, expanding activated CD4^+^ T cells expressing CCR5 at mucosal sites; this inadvertently created a target-rich environment for HIV, increasing susceptibility to infection rather than preventing it [17]. A corresponding trial in South Africa, Phambili (HVTN 503), using the same rAd5 vaccine, was halted when STEP’s results became known, and Phambili later showed no efficacy as well [4].

Despite these failures, STEP provided valuable scientific insights. Post hoc sieve analyses of viruses from infected participants showed the vaccine had exerted pressure on the virus’s gag sequence—viruses infecting vaccine recipients had more gag mutations in certain epitopes compared to those infecting placebo recipients [18]. This indicated that vaccine-induced T cells did recognize and impact the infecting virus, even if they did not prevent infection [18]. The STEP trial taught researchers that T cells alone, while important, were likely insufficient for protection, and that vector immunity and mucosal factors needed careful consideration in future designs [3,4].

### 4.3. RV144 Efficacy Trial in Thailand (2003–2009)

The RV144 trial in Thailand was a milestone in HIV vaccine research. The trial tested a prime-boost vaccine approach: participants first received a canarypox vector ALVAC-HIV (vCP1521) containing HIV-1 proteins (Env/gp120 from subtype E and Gag/Pol from subtype B), followed by a protein booster (AIDSVAX B/E) consisting of gp120 proteins from two HIV strains, combined with an alum adjuvant [19]. The initial vaccine was administered at 0, 1, 3, and 6 months to prime the immune system to activate CD8^+^ T cells and prepare the immune system to mount a rapid response in the event that it is exposed to HIV again. The boost vaccine was then given at 3 and 6 months, with the aim of producing antibodies that could bind to the virus and prevent entry into the host cell. RV144 demonstrated that taking these two vaccines could have a synergistic effect [20]. This regimen was given to 16,402 adults (general community, men and women) in Thailand in 2003–2006, and results were reported in 2009. RV144 demonstrated a modest but significant efficacy: 31.2% reduction in HIV infection risk (95% CI 1.1–52.1%; *p* = 0.04) over 3.5 years [21]. Specifically, 51 of 8197 vaccine recipients became infected versus 74 of 8198 placebo recipients [4]. While protection was modest and appeared to wane over time, the most benefit was seen in the first year after vaccination, and RV144 was the first trial to ever show any protective effect, providing proof-of-concept that an HIV vaccine is possible [4].

In-depth immune correlates analysis of RV144 yielded important clues. Vaccine recipients who did not get infected had, on average, higher blood levels of IgG antibodies targeting the V1V2 region of gp120, as measured by binding to a scaffolded V1V2 protein antigen [4]. Those with the highest anti-V1V2 IgG responses had the lowest infection rates, suggesting these antibodies may have contributed to protection [4]. Conversely, vaccine recipients with high levels of Env-specific IgA antibodies had less protection—high IgA appeared to mitigate the protective effect of IgG, perhaps by competing for the same Env epitopes or Fc receptors [4]. Given that the vaccine only showed partial protection from infection, neutralizing antibody responses in RV144 were weak and narrow, as expected since the immunogens were not designed to elicit bnAbs, and there was no correlation seen with neutralization of lab strains [4].

After RV144, researchers began focusing more on non-neutralizing antibodies that can trigger immune functions like antibody-dependent cellular cytotoxicity (ADCC)—a process where antibodies help immune cells kill HIV-infected cells [4]. RV144 participants produced antibodies capable of mediating ADCC, and this capability correlated with a reduced HIV infection risk, especially in the presence of low IgA [4]. Besides antibodies, the RV144 vaccine regimen also stimulated CD4^+^ T-cell responses in about half of the participants. The majority generated mostly T cells producing both IL-2 and IFN-γ, and a smaller proportion developed CD8^+^ T-cell responses [22]. Interestingly, polyfunctional CD4 T-cell responses showed a weak correlation with lower infection risk when interacting with the antibody responses [22]. The study also revealed that antibodies targeting a specific region of HIV (the V1V2 loop) were particularly important for protection, identifying a promising new target for future vaccines [19]. Encouraged by these findings, researchers aimed to further improve the RV144 vaccine approach by enhancing the magnitude and durability of V1V2-targeted antibodies, improving antibody breadth, and adapting the regimen for regions with different HIV subtypes.

### 4.4. DNA Prime–Ad5 Boost: The HVTN 505 Trial (2009–2013)

Another strategy evaluated in the late 2000s was a DNA prime followed by recombinant Ad5 boost, which sought to evaluate T cell response, much like the STEP and Phambili studies. However, it built upon the findings of RV144 by combining the safety and priming of DNA vaccines with the strong T-cell boost of a viral vector [2]. HVTN 505 trial was a Phase 2b clinical study conducted in the United States from 2009 until its early termination in 2013 [2]. It enrolled 2,504 participants, primarily men who have sex with men and transgender women [2]. The vaccine regimen involved two steps:

1. DNA Priming: Participants received three initial injections (at months 0, 1, and 2), each containing a mixture of six DNA plasmids encoding multiple HIV proteins: envelope glycoproteins from subtypes A, B, and C, as well as Gag, Pol, and Nef proteins [2].
2. Ad5 Boost: At month 6, participants received a booster vaccination using a recombinant Ad5 vector encoding HIV proteins (Gag-Pol from subtype B, and env glycoproteins from subtypes A, B, and C) [2].

Unlike the earlier STEP trial, this Ad5 vector included envelope proteins (Env) to stimulate both T-cell responses and antibody production. To avoid the Ad5 immunity problem seen in the STEP trial, the HVTN 505 only enrolled participants who were Ad5-seronegative, and most participants were predominantly circumcised men to mitigate potential risk factors. Despite these precautions, the interim results were disappointing: in April 2013, vaccinations were stopped due to a lack of efficacy [2]. The final analysis showed no efficacy—41 HIV infections occurred in the vaccine group vs. 30 in placebo (VE = −25%, *p* = 0.44) by 24 months (vaccine efficacy: −25%, not statistically significant) [2]. Additionally, the vaccine did not lower the viral load among participants who became infected [2].

However, there were some positive findings. Unlike the previous STEP trial, the vaccine did not increase the risk of HIV infection in any subgroup (unlike the earlier STEP trial) [2]. Additionally, it successfully generated immune responses, with over 70% of vaccinated participants producing binding antibodies to the Env antigens and about 50% demonstrating detectable CD4^+^ and CD8^+^ T-cell responses [2,23]. Notably, the Env antibodies induced were strain-specific and non-neutralizing, except against easy-to-neutralize lab strains [23]. Despite the lack of efficacy, a sieve analysis was conducted of breakthrough HIV-1 sequences from the HVTN 505 trial to identify potential pressures that may be involved in immune evasion [24]. This method can provide insight into differential vaccine efficacy based on the virus strain or post-acquisition effects that the vaccine may influence the evolution of the virus [24]. They found that vaccine recipients exhibited lower intra-host diversity sequence in gag, pol, and env-gp120 proteins, which were all included in the vaccine. Yet, they also found that Env-gp120 sequences in the vaccination group were significantly more distant compared to the subtype B Env sequence used in the vaccine. Overall, this analysis revealed genetic differences in the Env protein of breakthrough viruses between vaccine and placebo groups, suggesting vaccine-induced immune pressure on certain regions, specifically the Env-gp120 and CD4 antibody binding site [24]. However, this was not sufficient to impact overall infection rates.

The failure of HVTN 505, coming on the heels of RV144’s success, further emphasized that vaccine-induced responses must target the right viral vulnerabilities. Simply generating broad T-cell responses, such as those targeting Gag/Pol or Env-binding antibodies without neutralizing breadth, is insufficient to protect against HIV [2]. This trial emphasized the importance of generating antibodies with strong neutralizing abilities or potent effector functions and encouraged researchers to explore other vaccine vectors, adjuvants, and strategies for future HIV vaccines.

### 4.5. RV144 Follow-Up Trials in South Africa: HVTN 100 and HVTN 702 (2015–2020)

As vaccine development progressed, researchers continued to explore broadly neutralizing antibodies as a treatment, and there was also renewed interest in exploring the prime-boost strategy further. Building on the partial success of RV144, a series of trials were launched to increase the durability of protection and tailor the ALVAC/protein regimen to subtype C, the predominant HIV strain in southern Africa. Two key trials, HVTN 100 and HVTN 702, explored this approach [22,25].

HVTN 100 was a Phase 1b trial (2015) that took place in South Africa and tested a similar prime-boost to RV144. A prime vaccine, ALVAC-HIV (vCP2438), redesigned to encode subtype C Env (strain ZM96) and clade B Gag/Pol, was administered at 0, 1, 3, and 6 months [25,26]. This was followed by giving a booster vaccine consisting of an MF59 adjuvanted bivalent subtype C gp120 protein boost (strains TV1.C and 1086.C) at 3, 6, and 12 months [26]. The use of a different adjuvant compared to RV144 was in hopes of generating a more robust immune response [27]. HVTN 100 confirmed that this regimen was safe and elicited immune responses comparable to and stronger than those seen in RV144, with approximately 80% of participants developing high titers of V1V2-specific IgG antibodies and Env-binding IgG. Nonetheless, neutralizing activity was narrow and limited to tier 1 viruses only, and even though this trial did not demonstrate efficacy, it suggested that adding a booster vaccine after 12 months may increase the durability of the vaccine-induced immune response [25,26]. These promising results paved the way for the large-scale efficacy trial, HVTN 702 (Uhambo).

HVTN 702 (2016-2020) was a Phase 2b/3 efficacy trial conducted in South Africa starting in 2016, using the ALVAC (vCP2438) prime and MF59-adjuvanted bivalent subtype C gp120 boost, the same regimen as HVTN 100 but with an additional late boost to prolong immunity [22]. A total of 5,404 HIV-negative adults (both men and women at risk) were enrolled and randomized to vaccine or placebo [22]. Compared to the previous trial, the immunization schedule was modified and expanded by administering ALVAC-HIV primes at months 0 and 1, followed by ALVAC/gp120 boosts at months 3, 6, 12, and 18 (six shots total) [22]. The expectation was that the extra boosts at one year and 18 months might sustain high antibody levels.

Unfortunately, in early 2020, the trial’s Data Safety Monitoring Board determined the vaccine was not effective, and vaccinations were stopped for futility [28]. Final analysis showed no reduction in HIV infection in the vaccine group compared to the placebo [22]. Infection rates were essentially identical, and the cumulative infections by 24 months did not differ. The outcome of HVTN 702 was a stark failure despite being an optimized version of RV144, and the regimen had shown immunogenicity prior. The reasons are still being analyzed. Immune correlate studies presented in 2021 indicated that while the regimen induced robust CD4 T-cell responses that were higher than RV144, it elicited weaker V1V2-targeting antibody responses in this population [22]. In fact, only ~67% of vaccine recipients had IgG antibodies to the V1V2 loop (1086.C strain) two weeks after the final vaccination, compared to 100% in RV144, and the titers were lower [22].

The trial highlighted important lessons. First, the immunological targets validated by the RV144 trial—specifically V1V2-directed antibody responses—did not effectively translate across diverse populations or distinct HIV viral clades, particularly subtype C in southern Africa. Secondly, the reduced efficacy observed with MF59 compared to alum as an adjuvant underscores that adjuvant selection can markedly influence antibody specificity, magnitude, and functional quality. Moreover, simply increasing booster frequency was insufficient to compensate for underlying deficits in antibody quality. This finding reinforces the importance of generating antibodies with robust functional potency rather than merely maximizing antibody titers.

While the trial mirrors the immune correlates observed in RV144 by demonstrating that vaccine-induced V1V2-specific IgG and polyfunctional CD4^+^ T-cell responses continue to correlate with reduced HIV infection risk, their magnitude and quality must be significantly enhanced for meaningful protection [22]. Additionally, HIV incidence in the young women enrolled was very high, making vaccine efficacy detection harder [22].

Meanwhile, smaller studies were conducted concurrently to further improve the vaccine regimen, explore alternative priming methods and adjuvants, and guide future trials. These include the following:

HVTN 108 (Phase 1, 2015–2016) compared the alum vs. MF59 adjuvant with the subtype C ALVAC/gp120 regimen, finding that MF59 induced stronger Env-specific antibody responses and was therefore chosen for HVTN 702 [27].

HVTN 111 (Phase 1, 2015–2017) tested whether adding a DNA vaccine prime before protein could enhance responses. Volunteers received either DNA plasmid primes (encoding subtype C Env/Gag/Pol) followed by DNA/protein co-immunization boosts or the standard ALVAC prime/protein boost [25]. The results showed that the DNA prime was safe and that co-administration of DNA with the protein vaccine slightly increased V1V2-directed antibody response rate, potentially by providing additional antigenic stimulation to B cells [29]. However, the improvements were modest.

HVTN 120 (Phase 1/2a, 2017–2019) directly compared the MF59 and AS01ᴮ adjuvant in the protein boost, at two different protein doses [28]. The trial found that HVTN 120 found that the AS01ᴮ adjuvant significantly improved antibody and CD4^+^ T-cell responses, even at one-fifth the protein dose. It was demonstrated that the group that received 40 µg of gp120 with AS01ᴮ had higher antibody titers at 6 and 12 months compared to the group that received 200 µg with MF59 [28]. This suggests that future vaccine regimens might achieve better efficacy by using more powerful adjuvants like AS01ᴮ, which is used in shingles and malaria vaccines, to elicit stronger and more durable antibody responses.

### 4.6. “Mosaic” Vaccine Trials: Imbokodo and Mosaico (2017–2023)

In parallel to the RV144 line of research, Janssen/Johnson and Johnson led the “mosaic” vaccine approach, which aimed to address the extensive genetic diversity of HIV by designing antigens from computationally generated mosaic sequences that contain proteins from different HIV strains [30]. Mosaic-based adenovirus serotype 26 (Ad26) vectors combined with envelope glycoprotein boosts were initially promising in animal studies, protecting rhesus monkeys from simian–HIV (SHIV) challenges [30,31]. This concept, which seeks to trigger immune responses that provide protection against a broad range of HIV variants, was advanced into two large human efficacy trials: Imbokodo (HVTN 705/HPX2008) in sub-Saharan Africa and Mosaico (HVTN 706/HPX3002) in the Americas and Europe [30].

Beginning in 2017, Imbokodo enrolled 2,636 women in southern Africa who were at high risk of contracting HIV. The trial used a recombinant adenovirus serotype 26 vector expressing a mosaic of two Env and two Gag-Pol antigens (Ad26.Mos4.HIV) as the priming vaccine [30]. This was later followed by an alum adjuvanted protein boost vaccine expressing subtype C glycoprotein 140 (strain TV1), the soluble form of gp160, which is the precursor to glycoproteins 120 and 41 [30]. The priming vaccine was administered at 0, 3, 6, and 12 months, and the adjuvanted protein boost vaccine was administered at 6 and 12 months [30].

This trial concluded in 2021, and like previous trials, the vaccine was safe and induced immune responses, as indicated by the production of non-neutralizing antibodies against Env and T cell responses [30]. However, this vaccine did not significantly prevent HIV infection [30]. The trial primarily sought to assess HIV infection 7-24 months after vaccination, and findings demonstrated only 14% efficacy (95% CI: −22 to 40; *p* = 0.40) [30]. Therefore, the vaccine did not effectively lower HIV incidence when compared to the placebo group [31]. Despite this regimen demonstrating a 67% efficacy against SHIV challenges in animal studies, this protective effect did not translate to humans [30].

Mosaico was a Phase 3 trial built on a similar regimen, adjusted for a different population and slightly different envelope glycoprotein composition. It began in 2019 and enrolled about 3900 cisgender men and transgender individuals who have sex with men in the U.S., Latin America, and Europe [32]. The vaccine regimen in Mosaico used the same Ad26.Mos4.HIV vector prime at 0 and 3 months as Imbokodo, but the protein boosts at 6 and 12 months included a bivalent gp140 formulation. This included a subtype C gp140 (same as Imbokodo) plus a mosaic gp140 (designed to represent multiple subtypes), again adjuvanted with aluminum phosphate [33]. The addition of a mosaic Env protein in the boost regimen was intended to broaden antibody responses beyond subtype C.

In January 2023, an interim analysis revealed the vaccine was not effective, as the number of infections in the vaccine vs. placebo groups was essentially the same, and the DSMB recommended stopping the trial for futility [30,32]. No safety concerns were noted, but there was no protective efficacy evident, which prompted early termination [32]. It is also worth noting that participants were offered standard HIV prevention, including PrEP, which many took. This may have reduced the trial’s power to see an effect, but an effective vaccine should still have shown added protection in those who did not use PrEP [33].

Despite the broader immunogens, the Mosaico results mirrored the results from Imbokodo, and no overall reduction in HIV acquisition was observed [34]. Immunological analyses indicated that while the vaccine regimen elicited binding antibodies, these were primarily non-neutralizing, and the T-cell responses generated were weak [32]. Overall, the Imbokodo and Mosaico trials provided significant insight into vaccine development. They demonstrate that the current generation of mosaic-based vaccines is insufficient to overcome the diversity and evasion strategies of HIV. The concept of targeting multiple strains at once remains valid, and these trials emphasize the need for different vectors or more potent immunogens, such as inducing bnAbs, to generate different immune responses. Furthermore, the challenges of testing vaccines in the era of highly effective non-vaccine prevention were also highlighted. So, future trials will likely need to consider the use of PrEP, perhaps focusing on populations where PrEP uptake is lower or using efficacy endpoints that can still demonstrate additive benefit.

### 4.7. Passive Immunization Trials: The AMP Studies (2016–2021)

Broadly neutralizing antibodies (bnAbs) have emerged as promising agents to treat and prevent HIV infection [34]. They have a unique ability to neutralize a variety of HIV strains by targeting conserved viral sections of the envelope glycoprotein, such as gp120 and gp41, which are less prone to change [35]. Yet, differences in epitope availability and conformation may hinder the effectiveness of these versatile antibodies [36]. The primary mechanism by which they prevent HIV infection is by blocking the virus from entering the host cell. BnAbs can bind directly to gp120, which inhibits the ability to interact with the CD4 receptor on the host T-cell or through antibody-dependent cellular cytotoxicity [36]. Therefore, in addition to interfering with the viral entry process, bnAbs can also stimulate an immune system response by marking HIV-infected cells for destruction. Furthermore, bnAbs can also help control viral replication in individuals who are already infected with HIV by neutralizing circulating HIV and thus reducing the viral load [36].

While not testing a traditional vaccine, the Antibody Mediated Prevention (AMP) trials demonstrated the protective potential and limitations of broadly neutralizing antibodies (bnAbs) against HIV. The AMP studies included HVTN 703/HPTN 081 and HVTN 704/HPTN 085, which were Phase 2b trials conducted in parallel between 2016 and 2018. The HVTN 703/HPTN 081 enrolled women in sub-Saharan Africa, while the HVTN 704/HPTN 085 enrolled men who have sex with men and transgender individuals in the Americas and Europe [37]. In these studies, participants received an intravenous infusion of the bnAb VRC01, a monoclonal antibody that has been found to neutralize a wide range of subtype B and C strains in vitro and 90% of lab strains, every eight weeks over the course of 20 months for a total of ten infusions [38,39]. Preclinical in vitro studies demonstrated that VRC01 targets a conserved region within the gp120 glycoprotein to inhibit the virus from binding to the host CD4 receptor [40]. VRC01 had been shown to be safe and could moderately protect rhesus macaques from SHIV, so the question was, would passive transfer of this single bnAb prevent HIV in humans? [41].

Published results in 2021 showed that, overall, VRC01 did not significantly reduce HIV infection rates compared to placebo [38]. However, the viruses infecting participants were analyzed for sensitivity to VRC01 in vitro. This revealed that approximately 30% of circulating strains in the trial were inherently sensitive to the antibody, as defined by an IC_80_ below a certain threshold. Against those VRC01-sensitive viruses, the antibody was about 75% efficacious in preventing infection [42]. In contrast, however, most HIV strains that participants were exposed to within these regions were resistant to VRC01. Therefore, the antibody could not neutralize these strains effectively, and in regions where the virus was insensitive to VRC01, the vaccination and placebo groups were infected at equal rates [39]. In other words, VRC01 worked when the virus was susceptible, but most viruses had some resistance, so overall protection was null [39]. Thus, VRC01 provided proof-of-concept evidence that bnAbs can indeed protect humans from HIV, but it also proved that one antibody is not enough, given the viral diversity [39]. More importantly, however, the AMP trials provided a benchmark for required antibody titers and breadth. For example, they identified clear benchmarks for protective prevention efficacy. Protection against infection correlated with two factors—the antibody serum concentration and the neutralization sensitivity of the virus (IC_80_ value) [42]. Together, they predicted effective protection, which defines the specific neutralization titer needed for 90% protection and provides a valuable target for vaccine development [43].

The AMP findings have already catalyzed the design of next-generation passive immunization trials using combinations of broadly neutralizing antibodies. For example, new trials are testing mixtures of 2–3 bnAbs targeting different viral epitopes, such as the CD4 binding site, V3 glycan, and membrane-proximal external region, which are capable of neutralizing over 95% of global strains [44]. Furthermore, antibodies are being engineered for prolonged half-lives, reducing infusion frequency [45]. Overall, the role of passive antibody trials is complementary to vaccine development. Broadly neutralizing antibodies have been shown to be safe and may be a transient treatment for high-risk populations or in HIV-exposed infants via maternal delivery until a vaccine is available [46]. This promising new strategy reveals what an ideal vaccine-induced antibody response needs to look like, and if a vaccine can induce the same breadth and potency of a combination of bnAbs, it might achieve sterilizing immunity.

### 4.8. Recent Insights and Emerging Strategies

Despite multiple efficacy trial failures in the past decade, each has yielded insights that are driving new approaches in HIV vaccine development. Here, we summarize several emerging approaches driven by advances in immunology, structural biology, and vaccine technology, highlighting recent research and clinical progress.

1. Germline-Targeting and Sequential Immunogen Design: One of the most promising new paradigms is germline-targeting vaccination to elicit broadly neutralizing antibodies. The idea is to design a series of immunogens that stimulate rare naive B cells towards developing broadly neutralizing activity through several stages of maturation that include somatic hypermutation and affinity selection [47].

A breakthrough came from a Phase 1 trial (G001) conducted by the International AIDS Vaccine Initiative (IAVI) that evaluated the use of the germline-targeting immunogen eOD-GT8 60mer, an engineered outer domain of gp120 presented on a nanoparticle [48]. In 2021, it was reported that 97% of recipients who received eOD-GT8 developed detectable B-cells expressing VRC01-class precursors, the targeted bnAb germline [48]. Overall, this trial was successful in demonstrating that this immunogen could effectively prime the immune system. To build upon the success of the G001 trial, researchers began to consider enhancing these B cells by administering an additional tailored immunogen, with the hope of training them to recognize conserved sites of the native virus [48].

Trials are currently underway to test the effect of sequential immunizations in addition to germline-targeting immunogens. A Phase 1 trial (G002) is being conducted by IAVI to evaluate the safety and immunogenicity of boosting the immune system with a gp120 core-based nanoparticle immunogen, Core-g28v2 60mer [49,50]. A prior study on mouse models found that sequential immunization with a core-g28v2 60mer mRNA vaccine following an eOD-GT8 60mer prime led to the expansion of VRC01-class memory B cells and increased somatic hypermutation in the variable region of immunoglobulin genes [49]. The G002 trial is active currently, so comprehensive results are not available, but this study validates the eOD-GT8 60mer mRNA prime and core-g28v2 60mer mRNA boost regimen [49,50]. The goal is a vaccine regimen that will prime germline B cells to direct maturation toward producing broadly neutralizing antibodies that can provide protection against diverse HIV strains [51]. Furthermore, germline targeting is also being explored for bnAb classes aside from VRC01, such as those targeting the V3-glycan supersite on gp120 or the membrane-proximal external region of gp41 [51,52].

2. Epitope-Focused and Computationally Optimized Immunogens: To overcome the extensive genetic diversity and rapid mutation rate of HIV, scientists are developing immunogens that target specific vulnerable viral epitopes. For example, the V1V2 loop of the HIV envelope protein, which was associated with protection in the RV144 trial, is a promising target [21]. Scientists have isolated this loop and placed it on scaffold proteins, creating vaccines that specifically stimulate antibodies targeting this region [34]. In addition to focusing on the variable loops of envelope glycoprotein 120, the fusion peptide on gp4, which is essential for viral fusion with host cells, has also been recognized by several broadly neutralizing antibodies. Experimental vaccines presenting this fusion peptide linked to a carrier protein have successfully induced neutralizing antibodies in animal studies [52]. Additionally, to address the vast genetic diversity of HIV, mosaic Env trimers and center-of-tree (consensus) immunogens are being tested to broaden responses. “Mosaic” immunogens blend key genetic elements from diverse HIV strains into a single vaccine, encouraging the immune system to recognize common features across strains [53]. Similarly, “consensus” (center-of-tree) immunogens represent average sequences from multiple HIV variants to induce broader antibody responses [53].

Computational protein design has enabled the creation of nanoparticle-based vaccines. Nanoparticles can simultaneously display multiple different Env antigens, creating a “polyclonal mosaic” to the immune system [54]. The underlying insight is that to beat the diversity of HIV, vaccines may deliver many variants of the Env protein at once, or in sequence, to teach the immune system the commonalities. These novel immunogens are entering Phase 1 trials [53,54].

3. Improved Adjuvants and Delivery Systems: Adjuvants are critical components added to vaccines that facilitate antigen presentation to immune cells and therefore enhance the immune response [55]. As seen in HVTN 120, adjuvants can make a profound difference in the vaccine-induced immune response [28]. The success of the AS01ᴮ adjuvant in other vaccines, such as the malaria and shingles vaccines, has encouraged HIV vaccine trials to incorporate more potent adjuvants than previously used alum-based adjuvants or MF59 alone [55,56].

Future HIV vaccine strategies are exploring a variety of advanced adjuvants, including:

1. Saponin-based adjuvants, such as AS01 or Matrix-M, are known to strongly enhance antibody production and T-cell responses [55,57].
2. Toll-like receptor agonists, like 3M-052 (TLR7/8 agonist), can be formulated with alum to boost both antibody and T-cell immunity [58].
3. Novel cytokine adjuvants can be added to direct the immune response toward specific protective profiles [59].

There is also interest in mucosal adjuvants, like mucosal TLR ligands or CD40 agonists, designed specifically to enhance mucosal antibody IgA and tissue-resident T cells in the genital and rectal mucosa (Table 1), primary sites for HIV entry [60].

Moreover, vaccine delivery methods are being innovated to include needle-free injectors and electroporation that can improve the effectiveness of DNA-based vaccines by increasing their cellular uptake [60]. Persistent viral-vectored vectors, such as cytomegalovirus-based vectors, are also under exploration for their potential to continuously stimulate immunity [61]. Recombinant immune complex vaccines are also being explored to improve B-cell responses. These are pre-formed antigen–antibody complexes designed to deliver antigens directly to immune cells via Fc receptors, thereby enhancing B-cell responses [62].

4. mRNA Vaccine Technology: The rapid development and success of mRNA vaccines encapsulating lipid nanoparticles against SARS-CoV-2 has sparked interest in using mRNA technology to develop a safe and effective vaccine against HIV [63]. An mRNA vaccine is designed to activate the immune system and produce antibodies by using engineered messenger RNA that encodes a specific immunogen [63]. The two forms of RNA being explored are non-amplifying and self-amplifying mRNA vaccines [64]. The key distinction is that self-amplifying mRNA encodes information for the immunogen as well as proteins facilitating viral replication, which increases the amount of immunogen produced within the host [64]. In 2022, Moderna and IAVI collaborated to launch HVTN 302, a Phase I trial of three different stabilized HIV antigens [Table 2] [65]. This study was designed to evaluate the safety and immunogenicity of various non-amplifying mRNA vaccines, each encoding a different HIV-1 envelope (Env) protein [65,66].

Furthermore, nucleoside-modified mRNA is also being explored to further enhance the stability, efficiency, and effectiveness of mRNA vaccine administration [63]. Nucleosides can trigger the innate immune system, leading to an unwanted excess immune response and mistakenly causing the host to recognize the mRNA sequence as a threat [67]. Advancements in this area of study have demonstrated that nucleoside-modified mRNA formulations can induce robust germinal center responses, high antibody titers, and desirable T cell responses without excessive inflammation [68]. In general, the use of mRNA vaccines offers a few key benefits for HIV [Table 2]:

- Rapid and transient antigen expression: Because mRNA does not persist, it enables repeated vaccinations with evolving immunogens without concerns about anti-vector immunity that limit viral vector platforms [64].
- Complex antigen presentation: mRNA vaccines can produce membrane-bound or soluble forms of the HIV Env protein directly within cells, potentially improving recognition by B cells and promoting the development of neutralizing antibodies [64,68].
- Multivalent immunogen combinations: mRNA technology easily allows for encoding multiple HIV antigens simultaneously, potentially overcoming HIV’s extensive diversity and variability [64,68].

The main challenge is the very high bar for neutralizing antibody breadth, but some scientists believe mRNA is uniquely suited to tackle this by facilitating rapid iteration of immunogen designs and combination approaches [64]. Importantly, lessons from COVID-19 mRNA vaccine development, such as optimizing lipid nanoparticle formulations to enhance germinal center responses, minimizing excessive inflammation, and promoting T follicular helper cell activation, are actively informing current HIV mRNA vaccine research [63,68]. Early results from mRNA HIV vaccines are anticipated in the next couple of years, and they represent a promising path, especially for complex regimens like sequential immunizations needed for broadly neutralizing antibodies (Table 2).

5. Passive Immunization and Bi-specific Antibodies as Prevention Adjuncts: The broadly neutralizing antibody (bNAb) combination approach partly shifts some burden off vaccines (Table 3). For instance, if highly potent antibodies can be given intermittently, perhaps as subcutaneous injections of a monoclonal or via gene therapy with an Adeno-Associated Virus (AAV) vector, then they could provide protection during periods of high risk [69]. Some envision a future HIV prevention strategy where individuals receive an infusion of a tri-specific bnAb, in which one molecule can bind to three different critical sites on the envelope protein, alongside antiretroviral treatment or until they complete an active vaccine regimen [70]. Indeed, a tri-specific antibody binding to CD4 binding sites, membrane proximal external regions, and V1V2 glycan sites has been engineered and shown extraordinary breadth in vitro [69,70]. Clinical trials, however, are still needed to confirm its safety and real-world efficacy, although early results suggest significant potential as a preventive tool (Table 3).

Moreover, passive antibodies might be used to synergize with vaccines. For example, bnAbs administered alongside vaccines could temporarily block incoming HIV infections, effectively buying time while the vaccine-induced immunity develops or matures [71]. Additionally, studies in non-human primates have explored the concept of “immuno-focusing,” where sub-protective doses of bnAbs are administered together with vaccines to direct the immune response toward critical viral epitopes, helping shape and enhance protective antibody responses [72].

There is also exploration of vectored immunoprophylaxis using gene therapy vectors (like AAV) to deliver genes encoding bnAbs into muscle cells (Table 2). The cells then continuously produce protective antibodies, potentially providing long-term protection after a single administration [73]. One trial delivered an AAV vector encoding the bnAb PG9. It was safe, but antibody levels were low [73]. New vectors and enhanced synthetic proteins such as eCD4-Ig, a molecule combining parts of the CD4 receptor and antibody domains that strongly neutralizes HIV by preventing viral entry, are also being developed [74]. Preclinical studies have shown strong protection, and these constructs continue to be refined for higher potency and long-lasting expression [74].

These innovative approaches, while not vaccines in the classical sense, are important parts of the broader strategy to eradicate HIV. They highlight the lesson that multiple complementary tools may be required. A vaccine might reduce risk by 50%, an antibody could add another layer of protection, and oral PrEP could be a fallback, altogether driving new infections down to near zero.

In summary, recent HIV vaccine development efforts are marked by a convergence of advanced immunogen design, powerful new platforms, and a deeper immunological understanding of what it will take to induce protective immunity. The failure of past trials has clearly shown that incremental changes to old approaches, such as changing a viral subtype or adding one more boost, were not enough. As a result, the field is pivoting to fundamentally new ideas that include influencing B-cell evolution and engineering multi-specific bnAbs to achieve potency and widespread viral recognition.

Major HIV Vaccine Trials (1990–2025)

**Table 2:** The table below summarizes key trials of HIV vaccine or immunization strategies from 1990 to the present, including the vaccine components, platforms, adjuvants, trial phase/population, and efficacy or outcome.

Trial	Years	Vaccine Components and Platforms	Adjuvants	Population and Phase	Outcome/Efficacy
VAX004	1998–2003	Bivalent gp120 (subtype B/B) protein subunit	Alum	MSM, High-risk Women; Phase 3	Vaccine efficacy (VE) 6% (95% CI −17 to 24)—not significant.
VAX003	1998–2003	Bivalent gp120 (subtype B/E) protein subunit	Alum	Injection drug users; Phase 3	VE 0% (95% CI −7 to 6). Highlighted limited protection from single protein immunogens.
STEP (HVTN 502)	2004–2007	Recombinant Ad5 vector encoding gag, pol, nef	None	MSM; Phase 2b	VE −18% overall; HR 1.4 (Ad5-seropositive uncircumcised subgroup). Indicates possible increased risk in some Ad5-seropositive individuals.
Phambili (HVTN 503)	2007	Recombinant Ad5 vector encoding gag, pol, nef	None	General; Phase 2b	Halted due to STEP results; Interim VE −69% (wide CI)
RV144	2003–2009	Canarypox vector (ALVAC) prime; gp120 protein boost (AIDSVAX B/E)	Alum	General community; Phase 3	VE 31.2% (95% CI 1.1–52.1) at 42 months, Demonstrated importance of prime-boost approach.
HVTN 505	2009–2013	DNA plasmid prime (Env, Gag, Pol, Nef) and Ad5 vector boost (Env, Gag-Pol)	None	MSM, Trans women; Phase 2b	VE −25%; *p* = 0.44. Underscored limits of DNA/Ad5 platform for HIV prevention.
HVTN 100	2015	ALVAC-HIV (subtype C Env, clade B Gag/Pol) prime; gp120 protein boost	MF59	General; Phase 1b	Immunogenic. 80% V1V2-IgG responders; GMT 1:−6300, but neutralization limited to Tier-1 strains
HVTN 702 (Uhambo)	2016–2020	ALVAC-HIV subtype C prime; gp120 subtype C protein boost	MF59	Adults at risk; Phase 2b/3	HR 1.02 (95% CI 0.81–1.30) → 0% VE. Illustrated challenges in translating RV144 efficacy to different populations and HIV strains.
Imbokodo (HVTN 705)	2017–2021	Ad26 vector mosaic Env, Gag-Pol prime; subtype C gp140 protein boost	Alum	High-risk women; Phase 2b	VE 14% (95% CI −22 to 40) months 7–24. Highlighted difficulties in inducing protective immune responses against diverse HIV variants.
Mosaico (HVTN 706)	2019–2023	Ad26 vector mosaic Env, Gag-Pol prime; mosaic gp140 protein boost	Aluminum Phosphate	MSM, Transgender; Phase 3	Futility stop; VE ≈ 0%. Further indicated limitations of mosaic immunogen approaches.
AMP Studies (HVTN 703/704)	2016–2021	Passive immunization with VRC01 bnAb	None	General; Phase 2b	Overall VE 0%; 75% VE vs. VRC01-sensitive viruses (IC_80_ < 1 µg/mL), demonstrating strain specificity and need for bnAb combinations.
IAVI G001	2021	Germline-targeting eOD-GT8 60mer nanoparticle immunogen	None	General; Phase 1	97% (35/36) generated VRC01-class precursors Represents a promising new strategy for inducing bnAbs.
HVTN 302	2022–Present	mRNA vaccines encoding different stabilized HIV Env antigens	Lipid nanoparticles	General; Phase 1	Phase 1 safety; early data show >90% binding-IgG seroconversion by day 28

MSM: Men who have sex with men; bnAb: broadly neutralizing antibody; Prime boost: initial vaccination (prime) followed by later booster shots (boost) to enhance immune response; ALVAC: canarypox virus vector; Ad26/Ad5: adenovirus serotype 26/5 vectors.

**Table 3:** Immune mechanisms and antigenic targets of leading candidates.

Strategy/Platform	Key Antigen (s)	Primary Immune Goal	Representative Trials
Broadly neutralizing antibody (bnAb) induction	Conserved Env epitopes (CD4bs, V3-glycan, fusion peptide, MPER) presented as stabilized trimers, germline-targeting nanoparticles, or mRNA-encoded Env	Serum bnAb titers (ID50 ≥ 1:200 against global panel)	IAVI G001 (eOD-GT8), HVTN 302 (BG505 MD39 mRNA)
Non-neutralizing/Fc-effector antibodies	V1V2 loop, gp120 outer domain, gp41 HR2	ADCC, ADCP, complement; correlate seen in RV144	RV144 (ALVAC + gp120), HVTN 100/702
Polyfunctional CD4^+^/CD8^+^ T-cell vaccines	Conserved Gag, Pol, Nef, or HTI mosaics delivered by Ad26, MVA, DNA	Cytolytic T_RM, IFN-γ / IL-2 polyfunctionality	STEP, Phambili, Imbokodo, Mosaico
Mucosal-immunity approaches	Vaginal/nasal gp140, intranasal Env+TLR7/8, RhCMV vectors	Secretory IgA; genital/rectal T_RM	CN54 intravaginal Phase I; RhCMV/SIV macaque studies
Passive immunization (bnAb combinations)	VRC01, 10-1074, PGT121, tri-specific antibodies	Canarypox vector (ALVAC) prime; gp120 protein boost (AIDSVAX B/E)	AMP VRC01, CAPRISA-012 VRC07-523LS/CAP256V2LS
Vectored immunoprophylaxis	AAV-encoded bnAb genes (PG9, eCD4-Ig)	Immediate, strain-breadth neutralization (IC80 ≤ 1 µg/mL)	AAV-PG9 Phase 1 (Lancet HIV 2019)

## 5. Conclusions

Over 35 years of HIV vaccine research have yielded a mix of discouraging trial results and invaluable scientific insights. The cumulative evidence has clarified why a protective HIV vaccine is such a formidable challenge. Unlike many viral infections, HIV does not elicit sterilizing immunity naturally, as no recovered person exists whose immune response we can simply mimic. Early vaccine attempts lacked the breadth and potency needed to overcome HIV’s genomic diversity and rapid mutation. The past trials often generated either binding antibodies without sufficient neutralizing breadth (as in the gp120 subunit and RV144-derived regimens) or robust T-cell responses without complementary antibodies (as in the adenovirus vector trials), and neither approach alone provided durable protection. Notably, the partially efficacious RV144 regimen likely succeeded in large part due to an unusual antibody response (V1V2-targeting IgG) that current immunogens struggled to reproduce in other populations [22]. Moreover, that protection waned quickly, highlighting the insufficient durability of vaccine-induced immunity. Mucosal immunity has remained an inadequately achieved goal, demonstrated by most systemic vaccines eliciting poor mucosal IgA or tissue-resident T cells in the genital tract, yet these may be crucial for blocking sexual transmission at the portal of entry [60]. Finally, the capacity of HIV for immune evasion through glycan shielding of conserved sites, conformational masking of receptor-binding sites, and genetic mutation suggests that a successful vaccine must induce responses that target the virus where it cannot easily escape without a fitness cost. This likely entails targeting multiple vulnerable epitopes simultaneously through various approaches, such as broadly neutralizing antibodies to conserved Env sites and eliciting a strong T cell response to invariant internal proteins [35].

Despite the setbacks, the field has made remarkable progress in knowledge and tools. We now have a clear picture of the immune correlates that a vaccine should strive for, including high titers of broadly neutralizing antibodies or a combination of potent non-neutralizing antibody functions and T-cell responses that can synergize to prevent or control infection. We also have better immunogens and platforms to elicit these responses, from stabilized Env trimers that present neutralization epitopes in native form to mRNA delivery that can unleash iterative rounds of antigen exposure to adjuvants that induce germinal center reactions capable of maturing antibodies to high affinity.

The lessons learned from past trials are directly shaping future ones. For example, the failure of HVTN 702 taught us that simply increasing the frequency of boosting is not enough, and thus, qualitatively better antibodies, such as those targeting conserved V2 apex or V3-glycan sites with breadth, are still needed. The AMP trials taught us that a titer of ~50 µg/mL of a single bnAb like VRC01 is insufficient for most strains, and future vaccines will need to aim for either higher titer, greater breadth, or both, via multiple specificities [44]. Trials like HVTN 120 showed that the choice of adjuvant (AS01 vs. MF59) can be the difference between moderate and robust immunogenicity, guiding future protein-based vaccines to use the best adjuvants available [28]. The disappointments of mosaic trials are driving researchers to combine neutralizing antibody goals with mosaic T-cell approaches. In other words, future vaccines might use mosaic antigens to prime broad T-cell immunity and simultaneously include engineered Env immunogens to induce bnAbs.

Looking forward, several promising strategies are on the horizon. Sequential immunization schedules aimed at bnAbs could, if successful, finally crack the problem of inducing an immune response that combats the variability of HIV. Multivalent mRNA vaccines, for example, are vaccines designed to expose the immune system to multiple antigens from different viral strains and therefore train broad reactivity [68]. Additionally, passive immunoprophylaxis with bnAbs, either via periodic injections or encoded by gene therapy, may serve as a temporary solution in high-risk populations and as a complement to active vaccines [73]. Finally, another intriguing idea is combining vaccine-induced immunity with long-acting antiretrovirals [75]. For instance, an individual could receive an HIV vaccine series, and during the months it takes for immunity to develop, they could be covered by a long-acting PrEP drug or antibody. This kind of integrated prevention strategy acknowledges that no single tool may be completely effective, but together they could achieve strong, long-lasting protection.

However, there are significant challenges remaining. The ability of HIV to mutate means any vaccine-induced immunity will exert selection pressure, so researchers must anticipate escape mutants and design immunogens that either target critical regions of the virus to minimize escape or induce immune memory capable of adapting through continuous stimulation, as provided by replicating vectors. The hurdle of delivering vaccines to the mucosa might be addressed by novel routes like intravaginal rings releasing protein immunogens with adjuvant or oral vaccines targeting gut-associated lymphoid tissue. Finally, practical considerations such as manufacturing complex immunogens, ensuring safety in diverse populations, and maintaining adherence to multi-dose regimens may also require innovative solutions.

In conclusion, three decades of intensive research have taught us that an HIV vaccine will likely not resemble any traditional vaccine, as it may require novel antigen designs, delivery methods, and a combination of approaches. The road to an HIV vaccine has been longer and more tortuous than anticipated, but the knowledge and insight gained along the way have advanced our understanding of HIV, driving development efforts toward a safe, effective, and widely accessible vaccine against HIV infection. Each trial has been a stepping stone, revealing crucial information about which immune responses to target, what not to do in trial design, and how humans respond to these unique vaccines. Ongoing research remains active and continues to explore novel approaches, and these recent advances offer hope for progress in the fight against HIV.

## Figures and Tables

**Table 1:** Different types of adjuvants used in HIV vaccines.

Adjuvant	Composition/Type	Mechanism/Immune Enhancement	Key HIV-Vaccine Usage/Trial
Alum	Aluminum salts	Biases toward Th2; promotes binding-antibody responses	AIDSVAX gp120 trials VAX003, VAX004; protein boost of RV144 [21,22]
MF59	Oil-in-water squalene emulsion	↑ Env-specific IgG magnitude and breadth vs. alum	HVTN 100, HVTN 702, HVTN 120 [23,24]
AS01ᴮ	Liposome + MPL + QS-21 (saponin)	Strong germinal-center and Th1 response; higher binding Abs and CD4 T-cells	40 µg gp120 + AS01ᴮ outperformed 200 µg + MF59 in HVTN 120 [25]
Matrix-M	Saponin nanoparticle	↑ Tfh cells and neutralizing Abs (preclinical HIV Env nanoparticles)	Phase 1 Env-nanoparticle study NCT05414786 (ongoing) [26]
3M-052-AF	Synthetic TLR7/8 agonist (often alum/PLGA combo)	Durable Env-specific plasma cells; ↑ Tier-2 neutralizing titers in rhesus macaques	Planned first-in-human trial (IAVI H008) with HIV Env trimer [27]
Cytokine adjuvants	IL-7 or IL-15 super agonist (N-803)	Boosts cytotoxic memory CD8 T cells and NK activity	Being evaluated in therapeutic HIV-vaccine protocols [28]
Mucosal adjuvants	CD40 agonist peptides, CpG-ODN, chitosan/TLR ligands	Elicit mucosal IgA and tissue-resident T cells in genital/rectal tissue	Several Phase 1 mucosal-prime studies in preparation [29]

## Data Availability

Data are available in the references.

## Abbreviations

The following abbreviations are used in this manuscript:

AIDS	Acquired Immunodeficiency Syndrome
Alum	Aluminum hydroxide gel adjuvant
ALVAC	Canarypox virus vector
Ad26/Ad5	Adenovirus serotype 26/5 vectors
bnAb	Broadly neutralizing antibody
Env	Envelope
eOD-GT8	Engineered Outer Domain-Germline Targeting 8
Gag	Group-specific antigen
gp120	Glycoprotein 120
HIV	Human immunodeficiency virus
HVTN	HIV Vaccine Trials Network
IAVI	International AIDS Vaccine Initiative
LNP	Lipid nanoparticle
MF59	Oil-in-water emulsion adjuvant
MSM	Men who have sex with men
Nef	Negative factor
Pol	Polymerase
PrEP	Pre-exposure prophylaxis
VRC01	Vaccine Research Center 01 antibody (specific bnAb)
